# Supplementary material for: Zfhx4 regulates endochondral ossification as the transcriptional platform of Osterix in mice
Source: Commun Biol. 2021 Nov 3;4:1258. doi: 10.1038/s42003-021-02793-9 (PMC8566502; doi:10.1038/s42003-021-02793-9)
Supplement: Supplementary file 2 — Supplementary Information [file 42003_2021_2793_MOESM2_ESM.pdf]

Supplementary information

Supplementary materials and methods

Sequence of the probe for Southern blot analysis of *Zfhx4* floxed mice

5’-TGGAAGCAGGCCATCCTGAACTGAGTGAAGCTGAACTGCAGCAGCTCTATGC
CTCCTTGCCAATGAATGGTGAGCTCTGGGCAGAGAGTGAGACAATGACCCAGGAT
GACCATGGCATAGATCAGGAAATGGAGAGAGAATATGAGGTGGACCATGAAGGGA
AGGCAAGTCCTGTAGAGAGCGACAGCAGCTCTATCCCAGATGATCTGGGCTTGGA
ACCAAAGCGGACCTTACCTTTTAGAAAAGGACCCAACTTTACAATGGAAAAATTCCT
CGATCCATCTCGTCCTTACAAGTGTACAGTGTGTAAAGAGTCATTTACCCAAAAGAA
CATTCTCTTGGTCCATTATAATTCAGTGTCTCACTTGCATAAGTTGAAAAAGGTTTTG
CAAGAAGCCTCCAGTCCTGTCCCCCAGGAAGCCAATAGCAGCACAGATAATAAGC
CCTACAAGTGCAGCACCTGCAGTGTTGCATACAGCCAAAGCTCGACCTTGGAGAT
CCACATGAGATCAGTGCTCCACCAGACAAAGGCCAGGGCTGCAAAGCTGGAACC
AAGTAGACACCTACCCAGTGGTCATAGCATCACAGCT-3’

Target sequences of probes for ISH and WISH analyses of *Zfhx4*

For *Zfhx4*-1 probe

5’-TACCTCACTCAGTCTGCTCCCCTCCTCCTAACACCTCATCTACCTCGCCGTCT
GCAGCTTCTTCTAATAACACCTACCCTCATCTCTCTTGCTTCTCCATGAAGTCCTG
GCCTAATATCCTTTTCCAAGCATCCGCCAGGAAAGCTGCTTCGTCCCCCTCTTCTC
CTCCCTCCCTCTCCTTGCCTTCAACGGTTACCTCAAGTTTGTGCAGCACCTCAGG
GGTTCAAACCTCACTACCCACAGAAAGTTGTTCAGATGAGTCTGACAGTGAGCTG
AGCCAGAAGCTACAAGACTTAGATAATTCTTTGGAAGTGAAAGCTAAACCTGCTTC
TGGCCTAGATGGTAATTTCAATAGTGTCCGAATGGATATGTTCAGTGTGTAGGAGT
GAAGACAGGATCCCGTGCTTAAAAAAAAATTTAAAAATAAAAAATTTTTAAAAAAAG
ACTTTAACTGCAGTTCCAAAGCTTCTCTAACCCAAAAATTACAGTACCAAATGATTG
ACTCAGGATTGTTTTTCCCATATTGATATGCTGGCAATATAGGATGGTATGTGGTG
AAGAAGACTAATGCAGGTGGTTGAATGCGCTTGTAATATATGCTAAAATATGGAAA
AGGAAAAAAATATCTCACAAGTTCTTTGGGAACTTGTTTCAAGCCAAAAACTCTCA
AGACAGAAAATTGCACCTCAGCTGGGTTGATTTCAAAATGCCAGCATGGACAGT-3’

For *Zfhx4*-2 probe

5’-GTCCAAAAGCAATGATCGGCCTGGTCACAAGCGCTTCAGAACCCAGATGAGCA

ATCTTCAACTAAAGGTCCTCAAGGCTTGCTTCAGTGACTATCGGACTCCAACCATG
CAAGAATGTGAAATGCTGGGCAATGAGATTGGTCTGCCCAAACGAGTGGTGCAG
GTGTGGTTTCAGAATGCTAGGGCAAAGGAAAAGAAGTTTAAAATTAATATAGGAAA
GCCGTTCATGATCAATCAAAGTGGAACAGATGGTACTAAGCCAGAGTGTACCCTC
TGTGGGGTGAAGTACTCTGCCCGCTTGTCCATCCGAGATCATATTTTTTCCAAACA
GCATATTTCCAAAGTGAGGGAGACTGTGGGCAGTCAGCTTGACCGGGAGAAAGA
TTACCTTGCTCCTACCACCGTTCGACAGCTAATGGCCCAACAAGAACTGGACCGT
ATAAAGAAAGCTTCTGATGTGCTGGGCCTAACAGTACAGCAGCAAGGCATCACCG
ATAACTGCTCGCTGCATGGTATCAGCTTGCAAGCAGCCTACCCTGGGCTCCCTGG
CCTTCCTCCAGTCATTCTTCCTGGAATGAATGGCCCTTCCTCCTTGCCAGGATTTC
CACAAAATTCAAACACATTAACATCTCCGGGTACAGGTATGCTTGGGTTTCCTAGT
TCAGCTACTTCGTCTCCTGCCCTGTCTCTCAGCAGTGGCCCCACCAAATCTTTGCT
GCAGACTCCACCACCTCCACCACCACCTCCTCCTCCATCCTCTCTGTCAGGA
CAGCAGACCGAGCCACAGAACAAAGAATCGGAGAAAAAGCAAACTAAGCCAAACA
AGGTGAAAAAAATCAAGGAGGAGGAATCGGAGGCTATCAAACCCGAAAAGCACC
CCAAAAAGGAGGAAAAAATCTCATCTGCTCTTACAGTGTTGGGCAAAGTGGTAGG
GGAAACACACATGGATCCTACTCAGCTGCAGGCCTTGCAGAATGCAATTGCTGGG
GACCCAGCATCCTTTATAGGAGGGCAGTTCTTGCCATACTTTATCCCTGGGTTTGC
ATCCTACTTTTCACCTCAGCTCCCTGGAACAGTGCAGGGAGGCTACCTGCCACCA
ATCTGTGGCATGGAGAGTCTCTTTCCCTATGGTCCTGCAGTACCTCAGACACTGG
CGGGCCTGTCCCCAGGGGCGCTTCTACAGCAGTAC-3’

For *Zfhx4*-3 probe

5’-ACCTGATCCAACCTTCCGCGGTTTATGGAGCGCTTTTCATGTTGAAAACGGTGA
CTCTTTGCAGGCTGGCTTTGCCTTCTTGAAGGGAAGTGCAAGCCCTTCCAGCTCA
GCAGAACAGCCGCTGGGGATCACCCACATGCCAAAGGCTGAAGTGAACCTGGGG
GGGCTGTCTAGTTTAGTAGTGAACACCCCAATTACCTCCGTCTCCCTCAGCCACTT
ATCATCTGAGTCTAGCAAGATGTCAGAGAGCAAAGACCAAGAGAACAACTGTGAA
AGGCCAAAAGAAAGTACCATCTTACACCCGAATGTGGGGTGCCCTGTCAAAAGTG
AACCCACTGAGCCGGGAGATGAGGATGAAGAGGATGCATATTCCAATGAACTCGA
TGACGAGGAAGTATTAGGTGAACTCACAGATAGTATTGGTAATAAAGATTTCCCTC
TCTTAAACCAAAGCATTTCTCCTTTATCATCCAGTGTGCTAAAATTTATTGAAAAAG
GTACCTCGTCCTCCTCGGGGACTATTGCTGAA-3’

Whole mount *in situ* hybridization (WISH)

A digoxigenin (DIG)-labeled single-stranded *Zfhx4*-3 mRNA probe

(Supplementary information) was synthesized using a DIG RNA Labeling Kit (Roche, Basel, Switzerland). ICR mouse embryos (E12) fixed with 4% paraformaldehyde in PBS were hybridized and visualized as described previously[29].

Measurement of hypertrophic chondrocyte and calcified area of femurs of mice.

Hypertrophic chondrocyte area and calcified areas visualized by von Kossa staining were measured by Image J software (National Institutes of Health, Bethesda, MA, USA).

In vitro binding assay

Lysates of 293FT cells transfected with or without Flag-Zfhx4 were immunoprecipitated with an anti-Flag antibody and protein G-conjugated magnetic beads. Each immunoprecipitated protein was incubated with lysates of limb bud cells isolated from wildtype or *Osterix* KO littermates and then precipitated by a magnet. The proteins associated with protein G-conjugated magnetic beads were determined by western blotting using anti-Runx2 (Cell Signaling Technology, Danvers, MA, USA) or -Flag antibodies. Small amounts of immunoprecipitates were used as input.

Luciferase reporter assay

The 1, 5, and 10 kb regions of the *Mmp13* gene promoter were amplified by PCR using specific primer sets (Supplementary Table 1). Then, the 1 kb PCR product with incorporated KpnI and XhoI restriction enzyme sites was subcloned into a pGL4.10 luciferase reporter plasmid (Promega, Madison, WI, USA). Additionally, 5 and 10 kb PCR products were subcloned into the XhoI site of the pGL4.10 luciferase reporter plasmid using the In-Fusion HD cloning kit (Takara, Kyoto, Japan). SW1353 cells were transfected with these *Mmp13* luciferase constructs together with the empty vector or Flag-Zfhx4. To introduce Osterix or Runx2 into cells, they were infected with corresponding adenoviruses at 40 h after transfection and incubated for 48 h. Luciferase activity was determined using specific substrates and a GloMax® Luminometer (Promega).

Chromatin immunoprecipitation (ChIP) assay

ChIP analysis was performed using a truChIP Chromatin Shearing Kit (Covaris,

Woburn, MA, USA) in accordance with the manufacturer's instructions. Briefly, primary chondrocytes were cultured for 2 days and then washed with PBS. Chromatin was fixed with formaldehyde to crosslink proteins to the chromatin. Crosslinked chromatin was sonicated in a Covaris M220 (Covaris). Sonicated chromatin was incubated with an anti-Zfhx4 antibody (abcam, London, UK), anti-Runx2 antibody (Cell Signaling Technology), anti-Osterix antibody (abcam), or normal Rabbit IgG (Cell Signaling Technology), followed by immunoprecipitation with protein G-conjugated magnetic beads. Immunoprecipitates were amplified by PCR using primer sets specific for the *Mmp13* gene promoter (Supplementary Table 2).

Organ culture of the mouse maxilla

Maxillary explant specimens were microdissected to be free of the mandible, tongue, and brain tissues from E14.2 WT and Zfhx4$^{-/-}$ littermates. Explants were placed in a glass vial with 2.25 ml Dulbecco's modified Eagle's medium/Nutrient Mixture F-12 (Sigma-Aldrich) and 0.75 ml BGJb medium (Life Technologies), and cultured for 2 days at 37°C with 5% CO_2 using a rotary culture system. At the end of the 2 days of culture, the explants were fixed in 4% paraformaldehyde. The specimens were whole-mount stained with DAPI and imaged by fluorescence microscopy.

Immunohistochemical analysis for the palatal shelf

Samples fixed with 4% PFA in PBS were embedded in paraffin and cut into 7-μm-thick sections. The sections were subjected to immunohistochemical staining using an anti-cleaved caspase 3 antibody (Cell Signaling Technology) at a 1:200 (v/v) dilution. Alexa Fluor 555-conjugated anti-rabbit IgG was used at a 1:500 (v/v) dilution as the secondary antibody for visualization under a fluorescence microscope. Counterstaining was performed using DAPI.

Supplementary Table 1

Sequence of primer sets for *Mmp13* luciferase construct

1kb	5'-AGTGGTACCATTGAAGATTACAAAAAGACAGGCACT-3'	
	5'-TGACTCGAGAAAAGAGACCAAAATAACCA-3'	
5kb	5'-GCTCGCTAGCCTCGAGGGTACCAGGAGTGGGGTA-3'	
	5'-TCTTGATATCCTCGAGAGAAAAGAGACCAAAATAACC-3'	
10kb	5'-GCTCGCTAGCCTCGAGGTCAGGTGCAGGACTTTC-3'	
	5'-TCTTGATATCCTCGAGAGAAAAGAGACCAAAATAACC-3'	

Red color: Sequence matched to the *Mmp13* gene promoter

Black color: Sequence of region tagged for subcloning

Supplementary Table 2

Sequence of primer sets for *Mmp13* ChIP assay

Primer1	5'-CCATGGGGCTAGAAAGTTAGT-3'
	5'-TCATCCAAGCATTACAACTCTGA-3'
Primer2	5'-ACTAGCCTTGGACGACAGC-3'
	5'-AATGAGGGTCCTTGAAGCGA-3'
Primer3	5'-TGTGGCAGGACTCAATCATCT-3'
	5'-ATAGTGGGGAGAAGCAGCAG-3'
Primer4	5'-GGATTCTCACTGTTGCTGCT-3'
	5'-CCTAAGTGTGGTTTGTGGCA-3'
Primer5	5'-CTGCTGCTTCTCCCCACTAT-3'
	5'-AAGAAGAAGGTGGCCAGGAT-3'
Primer6	5'-TCCCTCAGATTCTGCCACAA-3'
	5'-CCTCTGCAAACACAAGGTCT-3'

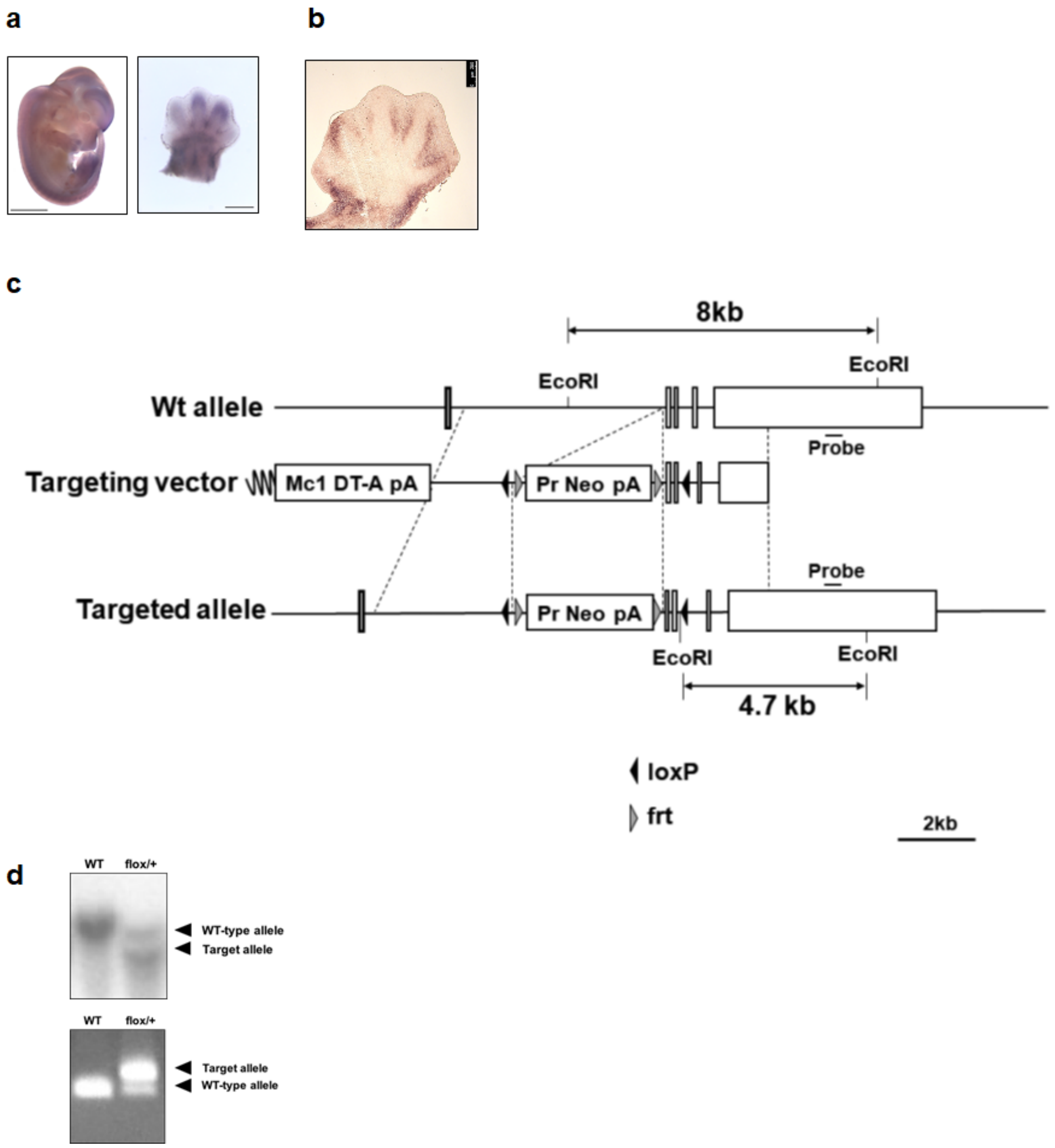

Supplementary Figure 1

Expression of *Zfhx4* in the limb bud and generation of *Zfhx4* floxed mice.

(a) Expression of *Zfhx4* in developing limbs of E12.5 mice determined by whole mount *in situ* hybridization. Scale bars = 2 mm (left panel) and 500 μm (right panel).

(b) Expression of *Zfhx4* in developing limbs of E12 mice determined by *in situ* hybridization. Scale bar = 250 μm

(c) Schematic diagram of *Zfhx4* floxed mice.

(d) Genotype analyses of *Zfhx4* floxed mice. Generation of heterozygous *Zfhx4* floxed mice was confirmed by Southern blotting (upper panel) and polymerase

chain reaction (lower panel).

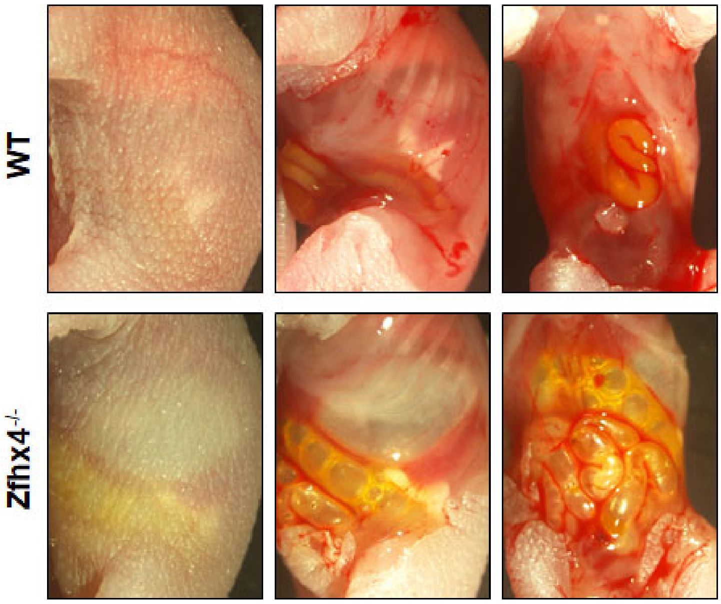

Supplementary Figure 2

Gastrointestinal tracts of *Zfhx4*$^{-/-}$ mice.

Photographs of entire gastrointestinal tracts of *Zfhx4*$^{-/-}$ mice, which were filled with air. WT: wild-type

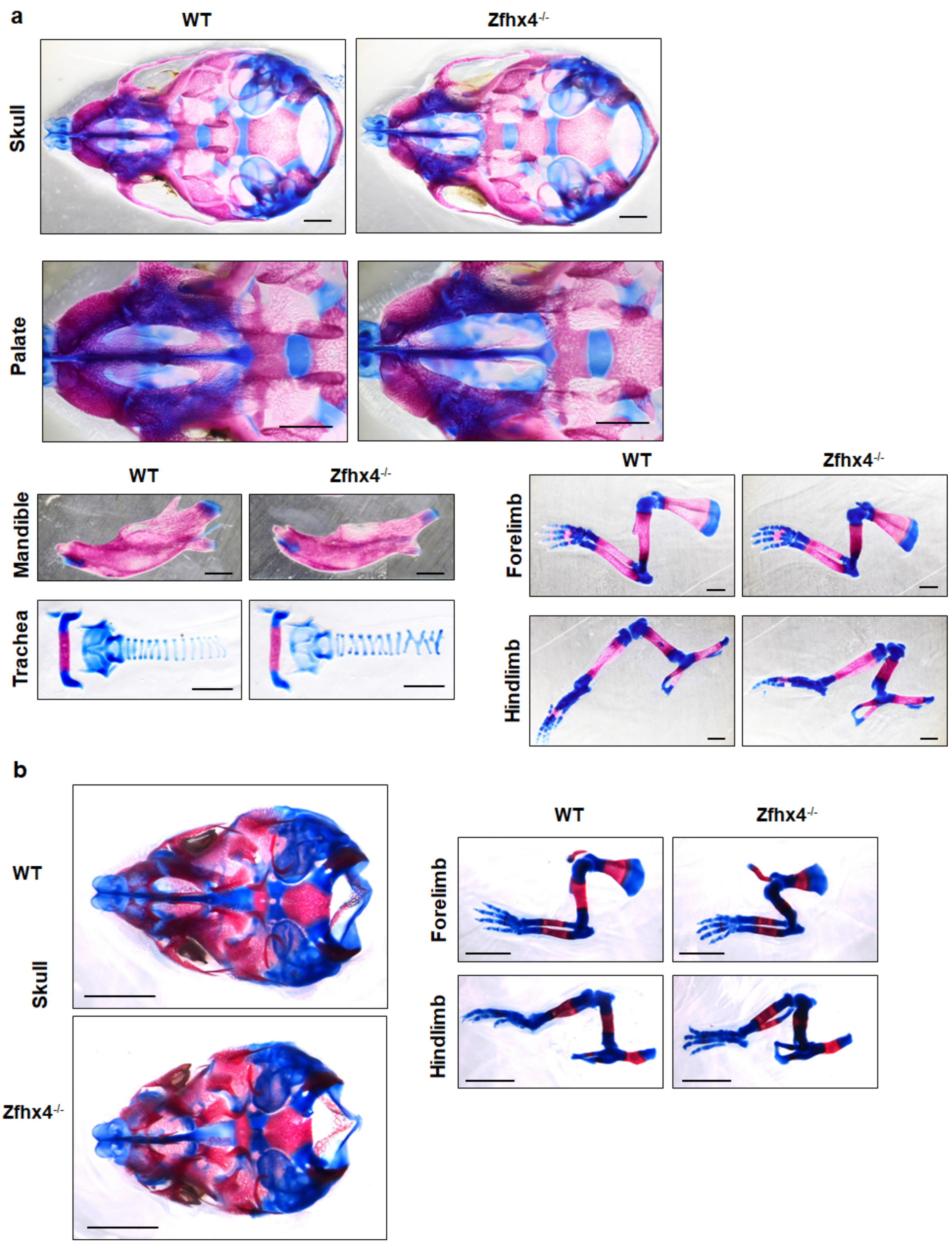

Supplementary Figure 3

Skeletal phenotypes of *Zfhx4*$^{-/-}$ mice.

(a) Microscopy images of skull, palate, mandible, trachea, forelimb, and hindlimb skeletal preparations stained with Alcian blue and alizarin red at P0. Wild-type (WT) and *Zfhx4*$^{-/-}$ littermates are shown. Scale bar = 1 mm.

(b) Microscopy images of skull, forelimb, and hindlimb skeletal preparations of E16.5 WT and *Zfhx4*$^{-/-}$ littermates stained with Alcian blue and alizarin red. Scale bar = 2 mm.

a

Genotype	WT	Zfhx4$^{+/-}$	Zfhx4$^{-/-}$
Number of pups	2	6	4
Dwarf pups (%)	0 (0%)	0 (0%)	4 (100%)

b

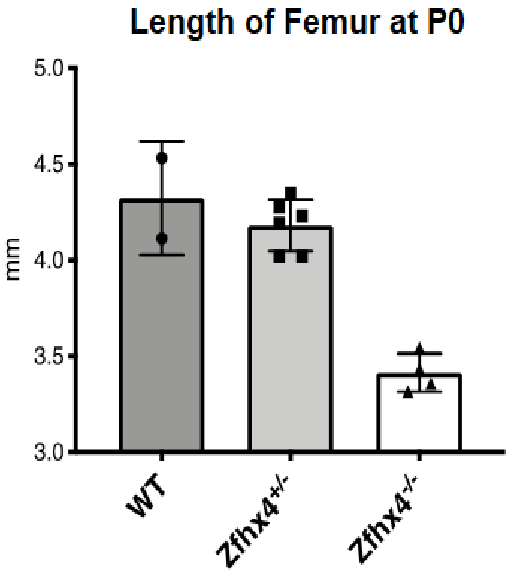

Supplementary Figure 4

Dwarf phenotypes of *Zfhx4*$^{-/-}$ mice.

(a) *Zfhx4*$^{+/-}$ mice were mated, and the dwarf phenotypes of the pups were examined at P0. WT: wild-type

(b) The length of femurs of these mice were measured after skeletal preparation. n: wild-type = 2, *Zfhx4*$^{+/-}$ = 6, *Zfhx4*$^{-/-}$ = 4

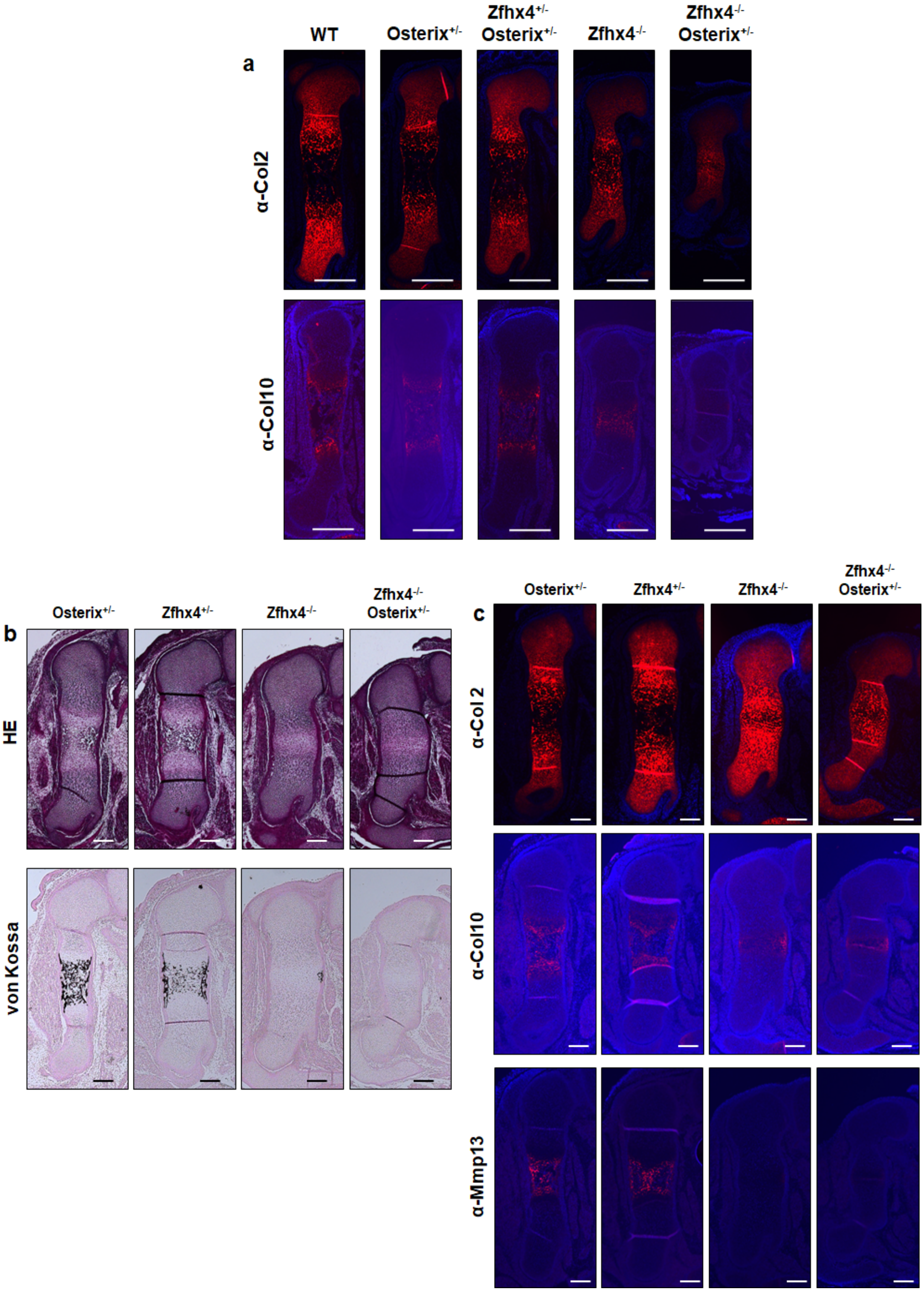

Supplementary Figure 5

Phenotypes of femurs of *Zfhx4*$^{-/-}$;*Osterix*$^{+/-}$ mice.

(a) Microscopy images of femurs of E16.5 wild-type (WT), *Osterix*$^{+/-}$, *Zfhx4*$^{+/-}$;*Osterix*$^{+/-}$; *Zfhx4*$^{-/-}$, and *Zfhx4*$^{-/-}$;*Osterix*$^{+/-}$ mice analyzed by

immunofluorescence staining with anti-Col2 and -Col10 antibodies. Scale bar = 500 μm.

(b) Microscopy images of femurs from E15.5 *Osterix*$^{+/-}$, *Zfhx4*$^{+/-}$, *Zfhx4*$^{-/-}$, and *Zfhx4*$^{-/-}$;*Osterix*$^{+/-}$ mice subjected to hematoxylin and eosin (HE) and von Kossa staining. Scale bar = 200 μm.

(c) Microscopy images of femurs from E15.5 *Osterix*$^{+/-}$, *Zfhx4*$^{+/-}$, *Zfhx4*$^{-/-}$, and *Zfhx4*$^{-/-}$;*Osterix*$^{+/-}$ mice determined by immunofluorescence staining with anti-Col2, -Col10, and -Mmp13 antibodies. Scale bar = 200 μm.

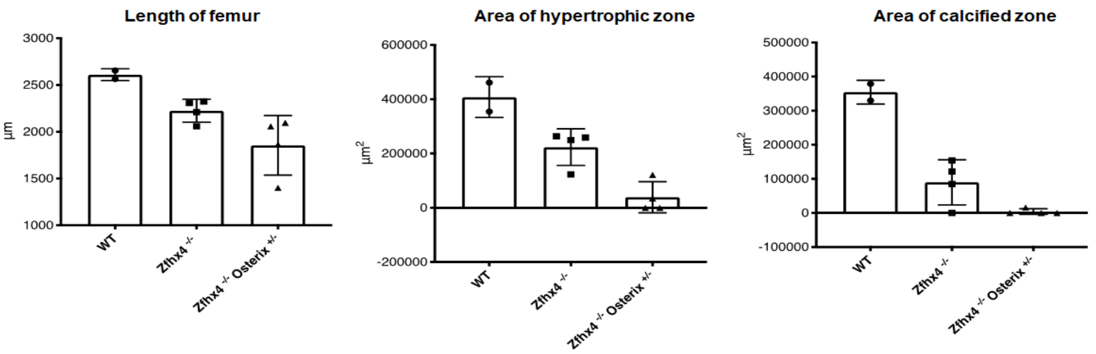

Supplementary Figure 6

Decreased hypertrophic and calcified area in *Zfhx4*$^{-/-}$;*Osterix*$^{+/-}$ mice.

Zfhx4$^{+/-}$ and *Zfhx4*$^{+/-}$;*Osterix*$^{+/-}$ mice were mated, and obtained aged-matched littermate embryos. The length and areas of hypertrophic and calcified zone of the femurs of the embryos were measured at E16.5. n: wild-type (WT) = 2, *Zfhx4*$^{-/-}$ = 4, *Zfhx4*$^{-/-}$;*Osterix*$^{+/-}$ = 4

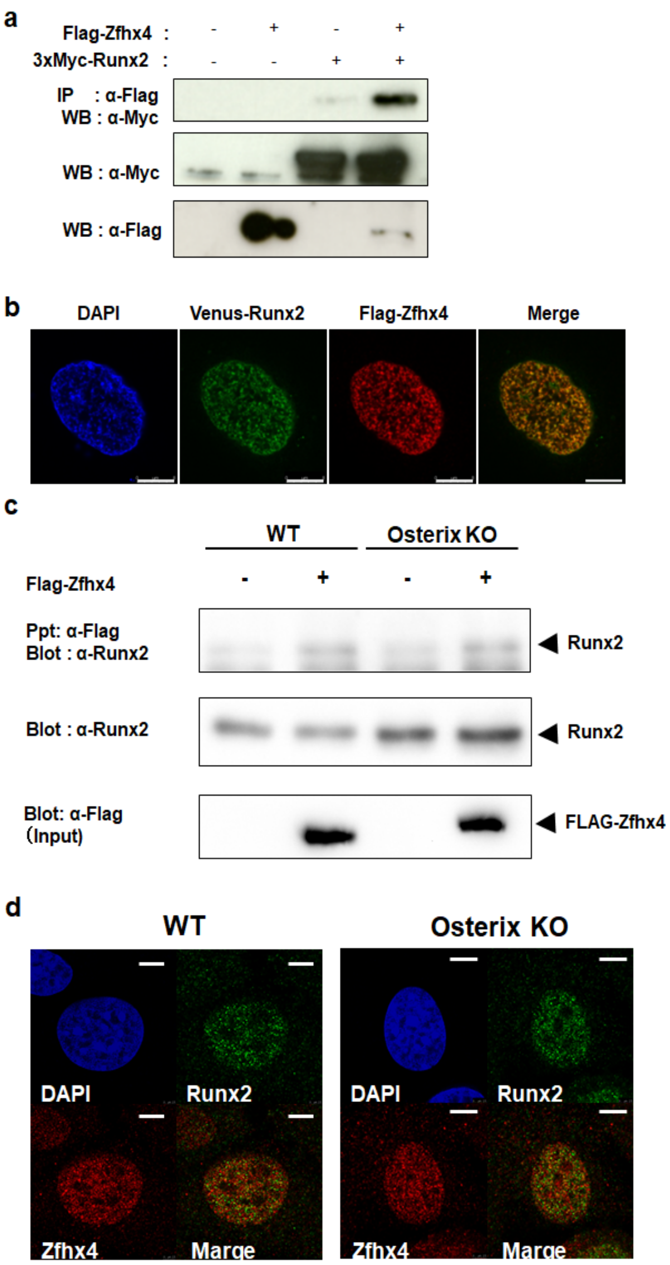

Supplementary Figure 7

Interaction of Zfhx4 with Runx2.

(a) Coimmunoprecipitation analysis of lysates of 293FT cells transfected with Flag-Zfhx4, 3xMyc-Runx2, or both. IP: immunoprecipitation; WB: western blotting.

(b) Colocalization of Flag-Zfhx4 (red) and Venus-Runx2 (green) in SW1353 cells. Scale bar = 8 μm.

(c) Association of Zfhx4 with Runx2 in WT and *Osterix* knockout (KO) mouse limb bud cells in vitro. Ppt: precipitation

(d) Colocalization of Zfhx4 and Runx2 in WT and *Osterix* KO mouse chondrocytes. Scale bar = 5 μm.

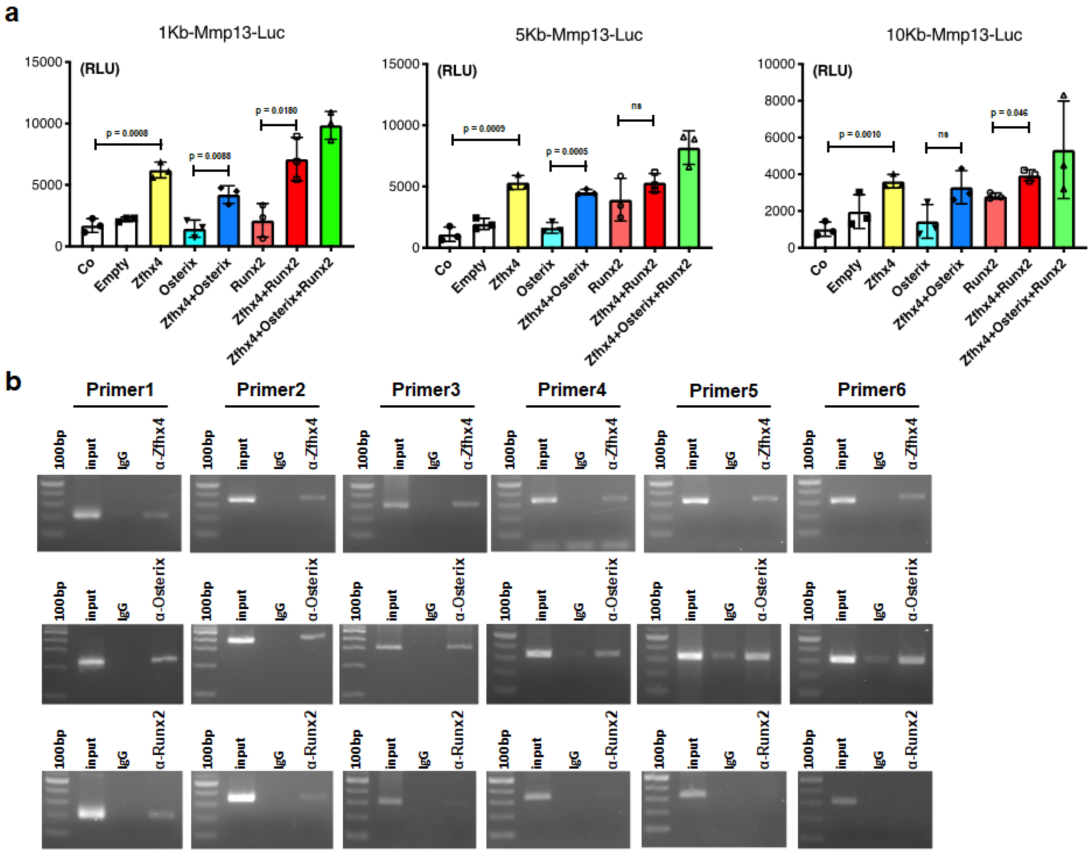

Supplementary Figure 8

Regulation of the *Mmp13* gene promoter by Zfhx4, Osterix, and Runx.

(a) Luciferase reporter assay using SW1353 cells transfected with 1, 5, or 10 kb *Mmp*13 gene luciferase promoter regions together with Zfhx4, Osterix, Runx2, or their combination as indicated.

(b) Chromatin immunoprecipitation analyses of mouse chondrocytes using anti-Zfhx4 (top), -Osterix (middle), or -Runx2 (bottom) antibodies. Binding to the *Mmp13* gene was amplified by primer sets as indicated.

a

Genotype	WT	Zfhx4$^{+/-}$	Zfhx4$^{-/-}$
Number of pups	23	30	25
Cleft palate pups (%)	0 (0%)	0 (0%)	25 (100%)

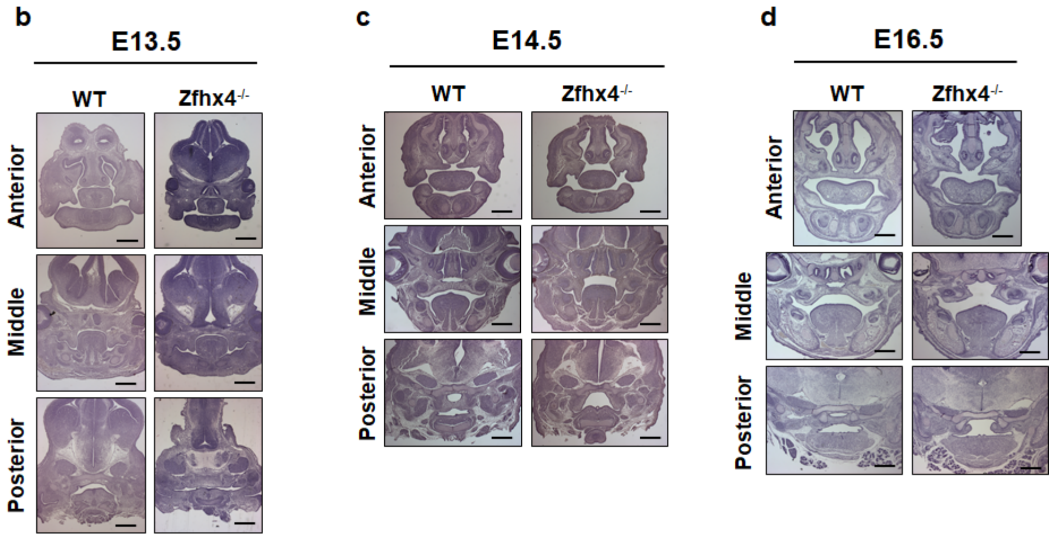

Supplementary Figure 9

Failure of elevation of palatal shelves in *Zfhx4*$^{-/-}$ mice.

(a) Penetrance of cleft palate in *Zfhx4*$^{-/-}$ mice.

(b–d) Lower magnification images of coronal sections from the anterior, middle, and posterior of crania of E13.5 (b), E14.5 (c), and E16.5 (d) wild-type (WT) and *Zfhx4*$^{-/-}$ littermates shown in Figure 4b. Scale bar = 500 μm.

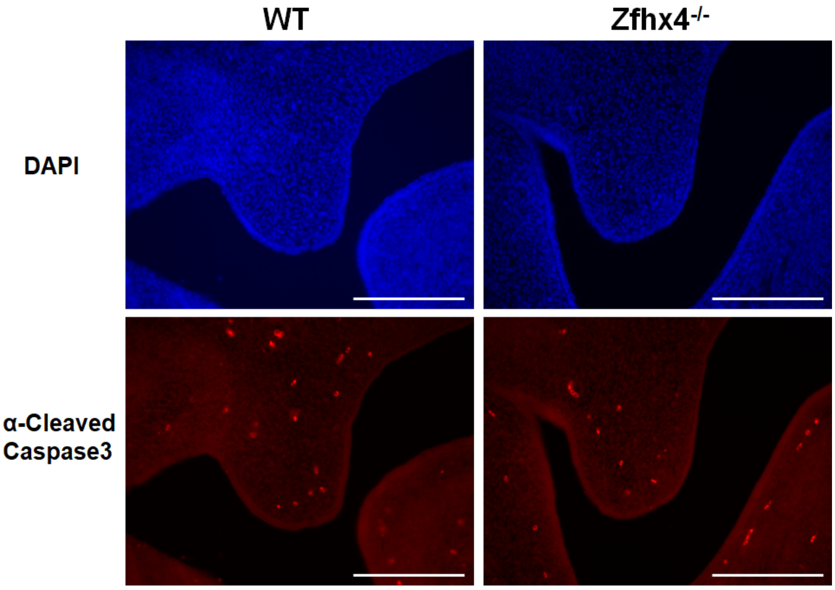

Supplementary Figure 10

No apoptotic state in the palatal shelf of *Zfhx4*$^{-/-}$ mice.

Immunofluorescence analyses of palatal shelves in E13.5 wild-type (WT) and *Zfhx4*$^{-/-}$ littermates using an anti-cleaved caspase 3 antibody. Scale bar = 200 μm.

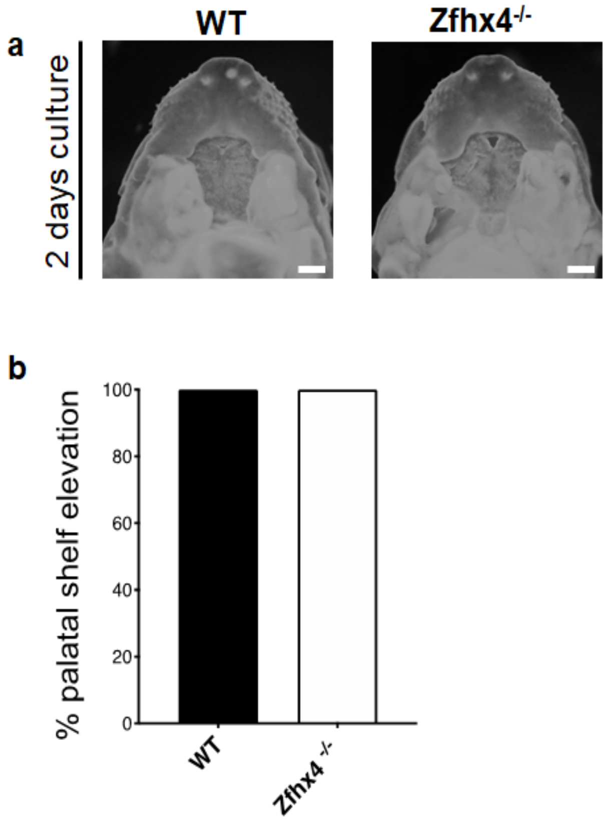

Supplementary Figure 11

Elevation of the palatal shelf of *Zfhx4*$^{-/-}$ mice ex vivo.

(a) Photograph of organ-cultured maxilla specimens isolated from E14.2 wild-type (WT) and *Zfhx4*$^{-/-}$ littermates. Scale bar = 500 μm.

(b) Frequency of palatal shelf elevation in (a). n = 6.

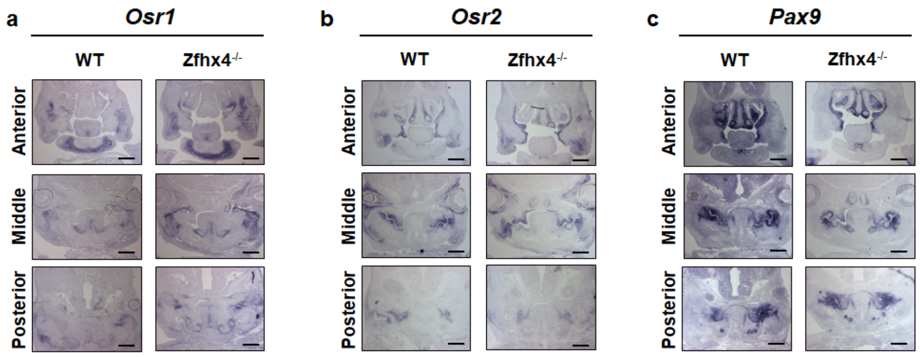

Supplementary Figure 12

Expression of *Osr1*, *Osr2*, and *Pax9* in *Zfhx4*$^{-/-}$ mice.

(a–c) Lower magnification images of coronal sections from anterior, middle, and posterior crania of E13.5 wild-type (WT) and *Zfhx4*$^{-/-}$ littermates shown in Figure 4f. Expression of *Osr1* (a), *Osr2* (b), and *Pax9* (c) determined by *in situ* hybridization. Scale bar = 500 μm.

a

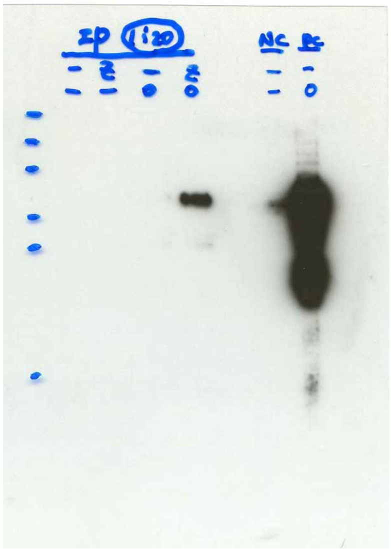

b

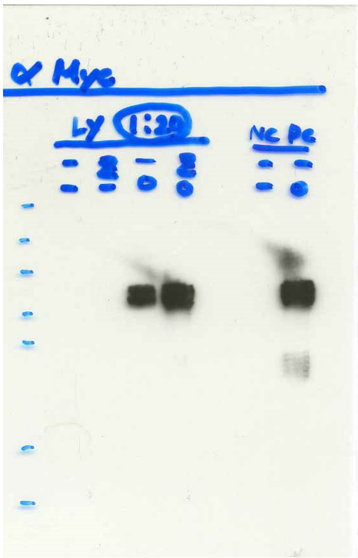

c

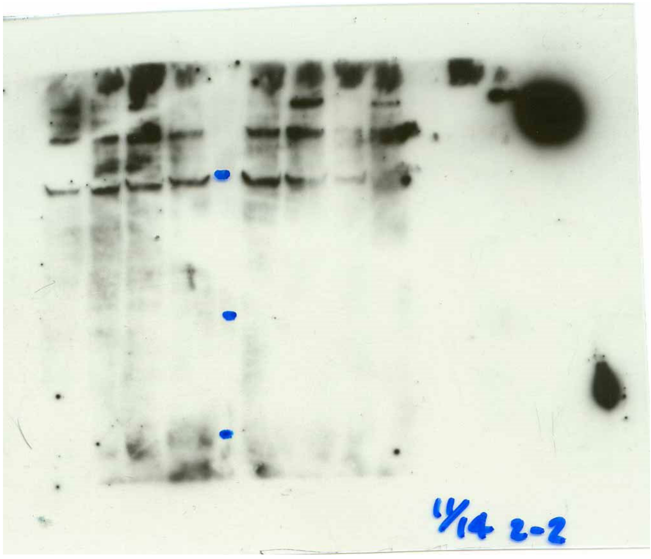

Supplementary Figure 13

The original uncropped images of Figure 3a. (a) the top panel of Figure 3a, (b) the middle panel of Figure 3a, and (c) the bottom panel of Figure 3a.
