# Supplementary material for: Zfhx4 regulates endochondral ossification as the transcriptional platform of Osterix in mice
Source: Commun Biol. 2021 Nov 3;4:1258. doi: 10.1038/s42003-021-02793-9 (PMC8566502; doi:10.1038/s42003-021-02793-9)
Supplement: Supplementary file 3 — Description of Additional Supplementary Files [file 42003_2021_2793_MOESM3_ESM.pdf]

Description of Additional Supplementary Files

File name: Supplementary Data 1.
Description: Source data for the graphs and charts in the main figures.
