# Supplementary material for: Zfhx4 regulates endochondral ossification as the transcriptional platform of Osterix in mice
Source: Commun Biol. 2021 Nov 3;4:1258. doi: 10.1038/s42003-021-02793-9 (PMC8566502; doi:10.1038/s42003-021-02793-9)
Supplement: Supplementary file 5 — Reporting Summary [file 42003_2021_2793_MOESM5_ESM.pdf]

nature portfolio

Corresponding author(s): Riko Nishimura
Eriko Nakamura

Last updated by author(s): Sep 10, 2021

Reporting Summary

Nature Portfolio wishes to improve the reproducibility of the work that we publish. This form provides structure for consistency and transparency in reporting. For further information on Nature Portfolio policies, see our Editorial Policies and the Editorial Policy Checklist.

Statistics

For all statistical analyses, confirm that the following items are present in the figure legend, table legend, main text, or Methods section.

n/a	Confirmed	
☐	☒	The exact sample size (n) for each experimental group/condition, given as a discrete number and unit of measurement
☐	☒	A statement on whether measurements were taken from distinct samples or whether the same sample was measured repeatedly
☐	☒	The statistical test(s) used AND whether they are one- or two-sided<br>*Only common tests should be described solely by name; describe more complex techniques in the Methods section.*
☒	☐	A description of all covariates tested
☒	☐	A description of any assumptions or corrections, such as tests of normality and adjustment for multiple comparisons
☐	☒	A full description of the statistical parameters including central tendency (e.g. means) or other basic estimates (e.g. regression coefficient) AND variation (e.g. standard deviation) or associated estimates of uncertainty (e.g. confidence intervals)
☐	☒	For null hypothesis testing, the test statistic (e.g. F, t, r) with confidence intervals, effect sizes, degrees of freedom and P value noted<br>*Give P values as exact values whenever suitable.*
☒	☐	For Bayesian analysis, information on the choice of priors and Markov chain Monte Carlo settings
☒	☐	For hierarchical and complex designs, identification of the appropriate level for tests and full reporting of outcomes
☒	☐	Estimates of effect sizes (e.g. Cohen's d, Pearson's r), indicating how they were calculated

Our web collection on statistics for biologists contains articles on many of the points above.

Software and code

Policy information about availability of computer code

Data collection	No software was used.
Data analysis	Statistic analyses were performed by GraphPad Prism software.

For manuscripts utilizing custom algorithms or software that are central to the research but not yet described in published literature, software must be made available to editors and reviewers. We strongly encourage code deposition in a community repository (e.g. GitHub). See the Nature Portfolio guidelines for submitting code & software for further information.

Data

Policy information about availability of data

All manuscripts must include a data availability statement. This statement should provide the following information, where applicable:

- Accession codes, unique identifiers, or web links for publicly available datasets
- A description of any restrictions on data availability
- For clinical datasets or third party data, please ensure that the statement adheres to our policy

All data that support the findings of this study are available from corresponding authors upon reasonable request.

Field-specific reporting

Life sciences study design

All studies must disclose on these points even when the disclosure is negative.

Sample size	No statistical methods were used to determine sample size.
Data exclusions	No data was excluded.
Replication	At least three times independent experiments were performed and reproducibility was confirmed.
Randomization	Not applicable.
Blinding	Investigators were not blinded.

Reporting for specific materials, systems and methods

We require information from authors about some types of materials, experimental systems and methods used in many studies. Here, indicate whether each material, system or method listed is relevant to your study. If you are not sure if a list item applies to your research, read the appropriate section before selecting a response.

Materials & experimental systems

n/a	Involved in the study
☐	☒ Antibodies
☐	☒ Eukaryotic cell lines
☒	☐ Palaeontology and archaeology
☐	☒ Animals and other organisms
☒	☐ Human research participants
☒	☐ Clinical data
☒	☐ Dual use research of concern

Methods

n/a	Involved in the study
☒	☐ ChIP-seq
☒	☐ Flow cytometry
☒	☐ MRI-based neuroimaging

Antibodies

Antibodies used	Col2 (7050, Chondrex), Col10 (LB0092, LSL), Mmp13 (ab39012, Abcam), Pcan (13119, Cell Signaling Technology), Ki67 (ab15580, Abcam), Flag (WAKO), Myc (ab9132, Abcam), Runx2 (12556, Cell Signaling Technology; D130-3. Medical and Biological Laboratories), Zfhx4 (ab254654, Abcam), Osterix (ab22552, Abcam), cleaved caspase3 (9661, Cell Signaling Technology)<br>Alexa Fluor 555-conjugated anti-rabbit IgG (A21429, Thermo Fisher Scientific), Alexa Fluor 555-conjugated anti-mouse IgG (A21424, Thermo Fisher Scientific), Alexa Fluor 488-conjugated anti-mouse IgG (A32723, Thermo Fisher Scientific)<br>horseradish peroxidase-conjugated anti-mouse antibody (Medical and Biological Laboratories), horseradish peroxidase-conjugated anti-rabbit antibody (Medical and Biological Laboratories), horseradish peroxidase-conjugated anti-goat antibody (Medical and Biological Laboratories)
Validation	All antibodies were validated by their manufactures.

Eukaryotic cell lines

Policy information about cell lines

Cell line source(s)	293, 293FT, SW1353
Authentication	None of the cell lines were authenticated.
Mycoplasma contamination	Cell lines were not tested for mycoplasma contamination.
Commonly misidentified lines (See ICLAC register)	No commonly misidentified line was used in this study.

Animals and other organisms

Policy information about studies involving animals; ARRIVE guidelines recommended for reporting animal research

Laboratory animals	C57BL6/J and ICR mice were purchased from Japan SLC (Shizuoka, Japan). Zfhx4 flox mice and Osterix flox were generated in collaboration of our laboratory and RIKEN Center for Biosystems Dynamics Research, and deposited to RIKEN Center. CAG-Cre transgenic mice were purchased from RIKEN BRC (Ibaraki, Japan).
Wild animals	N/A

Field-collected samples	N/A
Ethics oversight	All animal experiments were approved by the Osaka University Graduate School of Dentistry animal care committee and by the Institutional Animal Care and Use Committee (IACUC) of RIKEN Kobe Branch.

Note that full information on the approval of the study protocol must also be provided in the manuscript.
